# Chikungunya Virus Infection-related Rhabdomyolysis: A Case Report

**DOI:** 10.7759/cureus.4036

**Published:** 2019-02-08

**Authors:** Khaled Abdelmaqsoud Elfert, Mohammed Abdelwahed, Gerald Chi

**Affiliations:** 1 Internal Medicine, Hamad Medical Corporation, Doha, QAT; 2 Pathology, Boston University Medical Center, Boston, USA; 3 Internal Medicine, Beth Israel Deaconess Medical Center, Boston, USA

**Keywords:** chikungunya, rhabdomyolysis, virology

## Abstract

Chikungunya virus infection is an infection transmitted by mosquitoes. It classically presents with fever and arthralgia. The rhabdomyolysis as a complication of this infection is rarely reported in the literature. We report a case of a previously healthy 34-year-old male patient with acute onset of fever, myalgia, arthralgia, and dark colored urine. Laboratory values revealed elevated creatinine, positive urine dipstick for blood, but urine microscopy showed no red blood cells. Serum creatine kinase (CK) was elevated (>2000 U/L). Serology was positive for Chikungunya IgM antibodies. Rhabdomyolysis due to acute Chikungunya viral infection was diagnosed. Patient condition improved with supportive treatment and was discharged home.

## Introduction

Chikungunya virus is transmitted by the Aedes aegypti and albopictus, a mosquito species widely distributed in Asia, Europe, Africa, and America. Dengue and Zika viruses are transmitted by the same mosquito vectors as Chikungunya, and coinfections have been documented. Clinical manifestations include abrupt onset of fever, malaise, arthralgia, headache, and gastrointestinal manifestations. Few cases showed no symptoms [1-2].

Rhabdomyolysis is a rare complication of Chikungunya virus infection and only few reports are available about Chikungunya virus infection-related rhabdomyolysis [3-6]. In an outbreak of Chikungunya virus infection, one patient developed acute exacerbation of pre-existing heart failure, rhabdomyolysis, and multiple organ failure [3].

We report a case of Chikungunya virus infection resulting in rhabdomyolysis in an otherwise healthy patient with no pre-existing medical conditions.

## Case presentation

A 34-year-old Indian male patient presented with two days history of fever, myalgia, and arthralgia mainly in the shoulders, hips, and hands. The patient had travelled from India to Qatar six days prior to presenting to the hospital. He also complained of diarrhea (five watery stools per day) and dark colored urine. He had no history of sick contacts but reported mosquito exposure in India.

Review of systems revealed productive cough and eye redness, otherwise, it was unremarkable.

On initial examination, his oral temperature was 39.4°C, and blood pressure was 90/40 mmHg. There was conjunctival injection, and muscle tenderness in the shoulders and thigh muscles, but otherwise, the examination was normal.

The patient was resuscitated with five liters of normal saline and then blood pressure normalized.

Investigations

Laboratory investigations showed the following (Table 1):

**Table 1:** Laboratory investigations. This table shows the initial investigations and after four days of admission. N.B. His creatinine improved with fluid resuscitation.

	On admission	After four days	Normal ranges
Full blood count			
Hemoglobin	13.1 g/dL	13.9 g/dL	13- 17 g/dL
White blood cells	7.4 × 10^3^/μL	1.4 × 10^3 ^/μL	4.0-10.0 × 10^3^/μL
Absolute neutropenic count	6.2 × 10^3^/uL	0.5 × 10^3^/uL	2.0-7.0 × 10^3^/μL
Absolute lymphocytic count	0.9 × 10^3^/uL	0.7 × 10^3^/uL	1.0-3.0 × 10^3^/μL
Platelet count	137 × 10^3^ /μL	62 × 10^3^ /μL	150-400 × 10^3^/μL
General chemistry			
Urea	7.1 mmol/L	2.5 mmol/l	2.76-8.07 mmol/l
Creatinine	217 μmol/L	70 μmol/L	70-115 μmol/L
Corrected calcium	1.93 mmol/L		2.1-2.6 mmol/L
Creatine kinase	>2000 U/L		39-308 U/L

He had 3+ blood in the urine dipstick but urine microscopic analysis revealed no red blood cells. 

Differential diagnosis

The initial differential diagnosis included viral infection (e.g., including influenza, Dengue fever, Chikungunya virus infection) and parasitic infection (e.g., malaria). Bacterial infection was thought to be less likely because of lack of a focus of infection and symptoms (e.g., myalgias, arthralgias) pointing more towards a viral etiology.

Serologies for hepatitis C virus, hepatitis B virus, human immunodeficiency virus, and parvovirus; and serum polymerase chain reaction (PCR) for Epstein-Barr virus, cytomegalovirus, and adenovirus were negative. Blood smears for malarial parasites were negative. Dengue virus IgG was positive. However, IgM antibody was negative, suggesting past infection. Additionally, nasal swabs were sent for influenza, parainfluenza, corona viruses PCR and were all negative. Two sets of blood cultures came negative.

**Figure 1:**
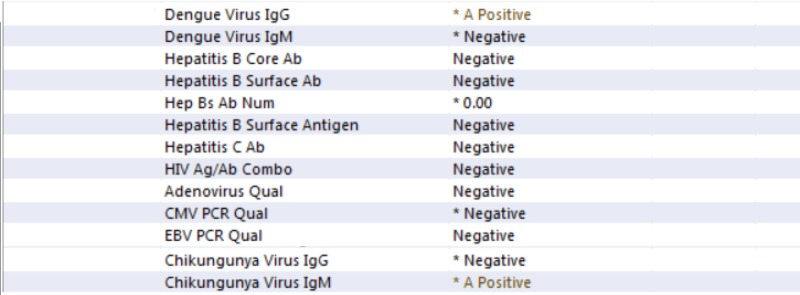
The viral panel. This image shows the viral panel which was sent for the patient. It shows that IgM for Chikungunya and IgG for dengue were positive.

Blood for Chikungunya IgM antibody test came positive, while IgG antibody test was negative suggesting acute infection (Figure 1) [1]. 

Treatment

The patient was given supportive treatment, and over his hospital stay, his symptoms improved and the laboratory abnormalities started to improve. He was discharged to home after six days.

## Discussion

We present a case of Chikungunya virus infection complicated by rhabdomyolysis, a rare complication of the infection. The patient recovered uneventfully with conservative management and was discharged to home.

The most common presenting features reported in the literature are fever (92%) which varies from low grade to high grade, and arthralgia (87%) which can occur without a fever. The joint pain is usually worse in the morning. It is relieved by mild exercise and aggravated by aggressive movements. Headache and chills occur in 62% of patients [7]. The diagnosis of Chikungunya fever is confirmed using real-time reverse-transcription polymerase chain reaction (RT-PCR) or Chikungunya virus serology [1].

Rhabdomyolysis is diagnosed in a patient with either an acute neuromuscular disease or dark urine without other symptoms when creatine kinase (CK) is markedly elevated. That elevation is typically five times the upper limit of normal and is usually above 5000 U/L [8]. So, usually, the patient will present with the classic triad of myalgias, generalized weakness, and darkened urine. On the other hand, asymptomatic serum CK elevation occurs with no or insignificant muscle-related signs and symptoms. In our case, the patient had myalgia and dark colored urine. In addition, he had a CK level of more than 2000 Ul/L, but we could not get the exact number as the Analytical Measuring Range of the analyzer was verified up to 2000 U/L only. The patient was not taking any medications prior to his current illness. He denied alcohol intake. He had no recent seizure episode or trauma. So, there was no other possible etiology to explain the rhabdomyolysis. 

Our literature search revealed a reported case of culture-confirmed Chikungunya virus infection-associated death in a patient who developed an acute exacerbation of pre-existing heart failure, rhabdomyolysis [3]. In another case report, a patient infected with Chikungunya virus developed rhabdomyolysis complicated by renal failure requiring dialysis [5]. In Suriname, during an outbreak of Chikungunya virus infection, a case of an 11-year old patient who presented with fever, myalgia, and dark colored urine was diagnosed with Chikungunya complicated with rhabdomyolysis due to viral myositis [6]. In one outbreak on Reunion Island, 15 out of the 157 infected patients had a CK level of more than 1000 U/L [9]. However, it is unclear how many of these patients had rhabdomyolysis vs. asymptomatic elevation of CK, as no patients were labeled as having rhabdomyolysis in this study and all we have are the laboratory values of the studied subjects. Two cross-sectional studies of outbreaks occurred in Malaysia and Southern Mexico did not report CK values for patients with Chikungunya virus infection [10-11]. A recent cross-sectional study, published in December 2018, studied the outbreak which occurred in French Guiana between February 2014 and October 2015; it showed that three patients out of the study population (*n* = 285) developed rhabdomyolysis [12].

Following the outbreak of Chikungunya virus in La Reunion islands in 2005, a number of studies started discussing the role of viral infection in affecting muscle tissues, causing rhabdomyolysis. A recent study discussed the role of attacking muscle satellite cells in the incidence of rhabdomyolysis. According to the study published by Ozden et al. [4], “Immunohistology on muscle biopsies from two Chikungunnya virus-infected patients with myositic syndrome showed that viral antigens were found exclusively inside skeletal muscle progenitor cells (designated as satellite cells), and not in muscle fibers.” Satellite cells are myogenic precursor cells that persist in postnatal and adult muscle. Also in vitro studies showed that the Chikungunya virus has the ability to replicate inside human satellite cells with cytopathic effect, while it is not growing inside the myotubes [4].

Regarding the hematological abnormalities, our patient developed neutropenia, lymphopenia, and thrombocytopenia. During an outbreak of Chikungunya infection in Malaysia, lymphopenia and neutropenia (counts <1 × 103 cells/mm3 ) were seen in two-thirds of presenting patients during their admission to the hospital (*n* = 117) with median lymphocyte and neutrophil count of 0.8 × 103 /uL and 0.9 × 103 /uL cells/mm^3^, respectively. Thrombocytopenia were seen in 89% of patients (platelets <150,000 cells/mm3) [10].

Blood chemistry for our patient showed he has hypocalcemia. Hypocalcemia is a laboratory abnormality known in patients with Chikungunya virus infection [11]. In an outbreak of Chikungunya on Reunion Island, hypocalcemia (blood calcium level, <2.25 mmol/L) was present in 86 patients out of 157 [9]. Hypocalcemia in rhabdomyolysis usually occurs in the first few days and is most likely attributed to the deposition of calcium in injured tissues [13]. As the hypocalcemia in Chikungunya virus infection is fairly common, while rhabdomyolysis is a rare complication of the infection, there could be other possible mechanisms for hypocalcemia other than muscle injury and rhabdomyolysis.

Regarding treatment of the acute phase, there is no specific antiviral therapy for acute Chikungunya virus infection. Treatment during the acute phase of the disease is symptomatic for fever and pain using nonsteroidal anti-inflammatory drugs. Systemic glucocorticoids and other immunosuppressive medications are better to be avoided in patients during acute infection [14].

## Conclusions

Physicians should be aware that rhabdomyolysis could be a part of Chikungunya virus infection presentation, which necessitates early and aggressive fluid resuscitation.
